# Supplementary figures and images for: Pre-existing heterosubtypic immunity provides a barrier to airborne transmission of influenza viruses
Source: PLoS Pathog. 2021 Feb 18;17(2):e1009273. doi: 10.1371/journal.ppat.1009273 (PMC7891786; doi:10.1371/journal.ppat.1009273)

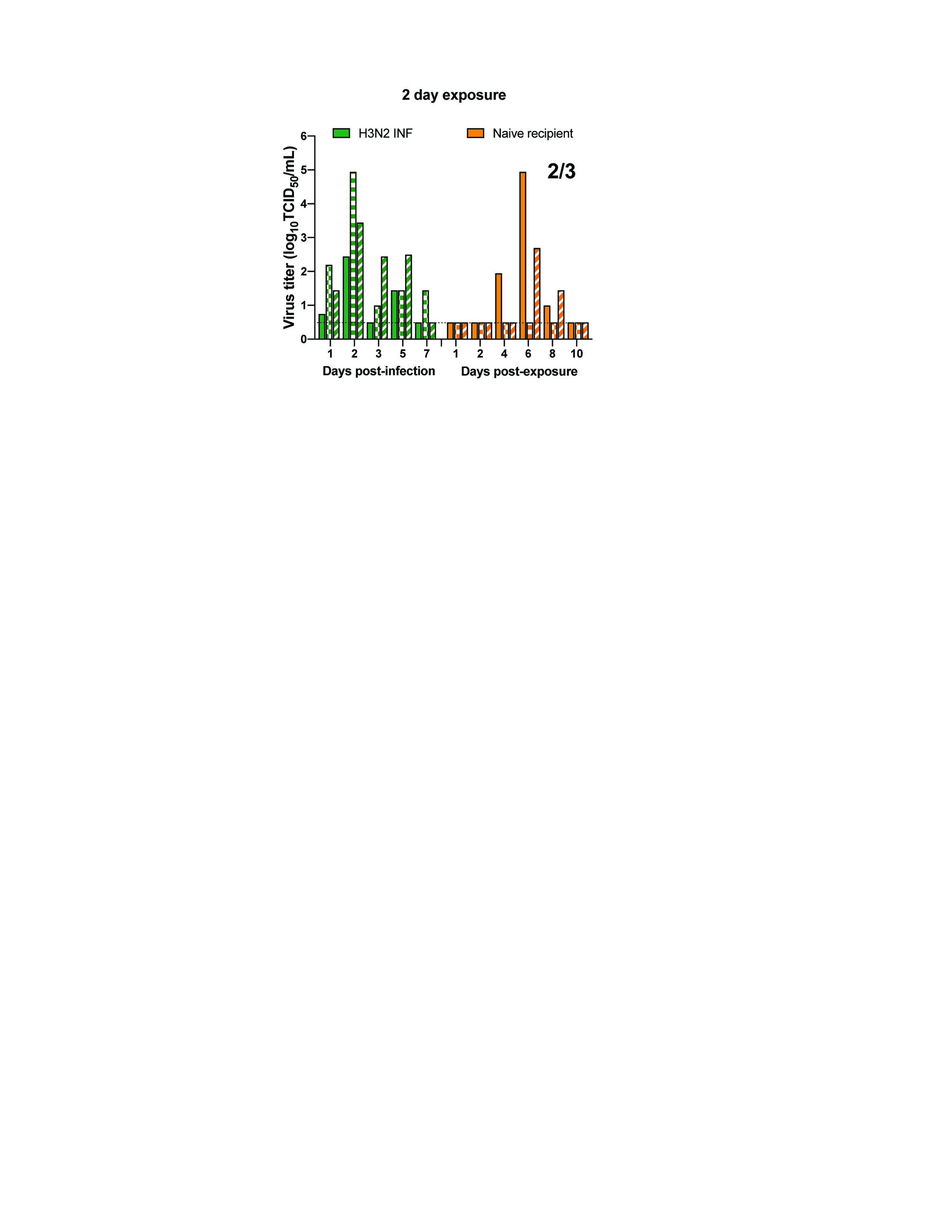

Supplement: S1 Fig — Three ferrets were infected with A/Perth/16/2009 (H3N2) and nasal washes were collected from each ferret on the indicated days post-infection. A naïve ferret was placed in the adjacent cage at 24 hour post-infection for 2 days and nasal washes were collected from each recipient ferret on the indicated days post-exposure. Bars indicate individual ferrets. All ferrets were serologically negative for circulating influenza viruses at the beginning of the study. The limit of detection was 100.5 TCID50/mL. TCID50, 50% tissue culture infectious dose. The viral titer data for 3 donor animals was previously published in (45). (TIF) [file ppat.1009273.s001.tif]

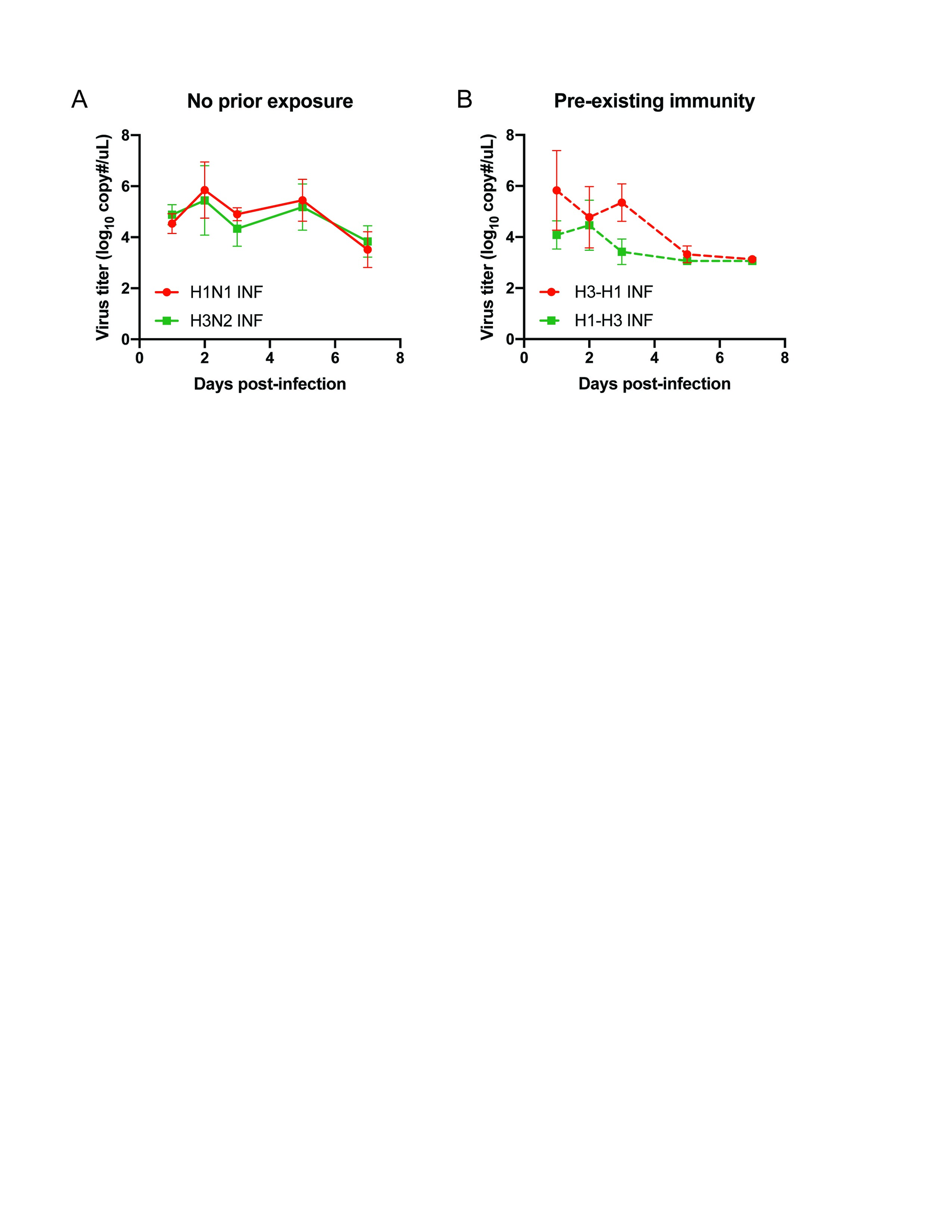

Supplement: S2 Fig — RNA was isolated from nasal wash samples at each of the indicated days post-infection from ferrets infected with H1N1pdm09 (red line) and H3N2 (green line). Data are shown as mean +/- SEM for 3–4 ferrets per condition. (TIF) [file ppat.1009273.s002.tif]

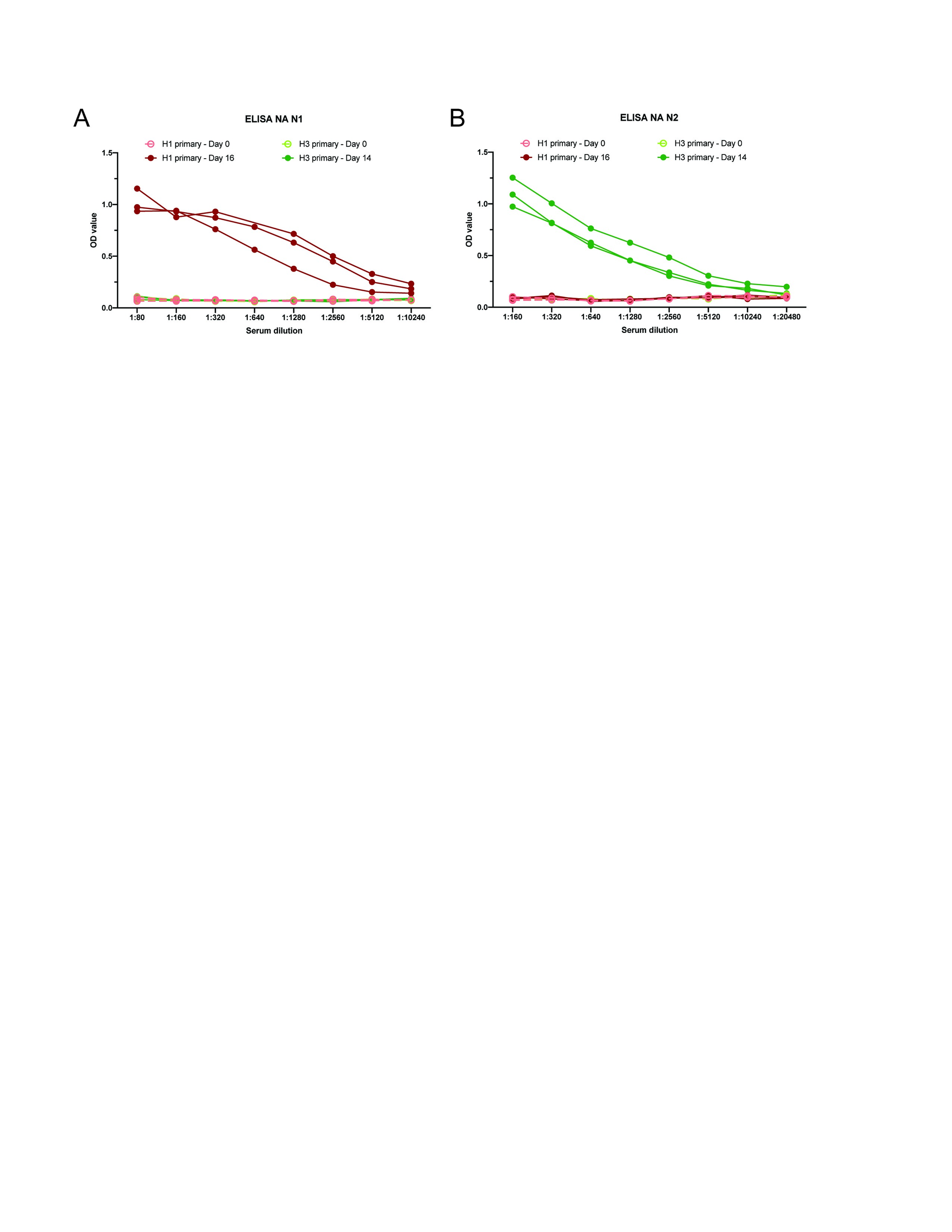

Supplement: S3 Fig — Ferrets were infected with either H1N1pdm09 (red) or H3N2 (green) and NA antibody levels were determined by ELISA using (A) recombinant A/California/07/2009 (H1N1)pdm09 N1 or (B) recombinant A/Brisbane/10/2007 N2 proteins. OD values for day 0 and day 14 or day 16 serum are displayed and each line indicates an individual ferret. Day 0 serum is presented in a lighter shade and with open circles, while day 14 or day 16 serum is a darker shade and solid circles. (TIF) [file ppat.1009273.s003.tif]

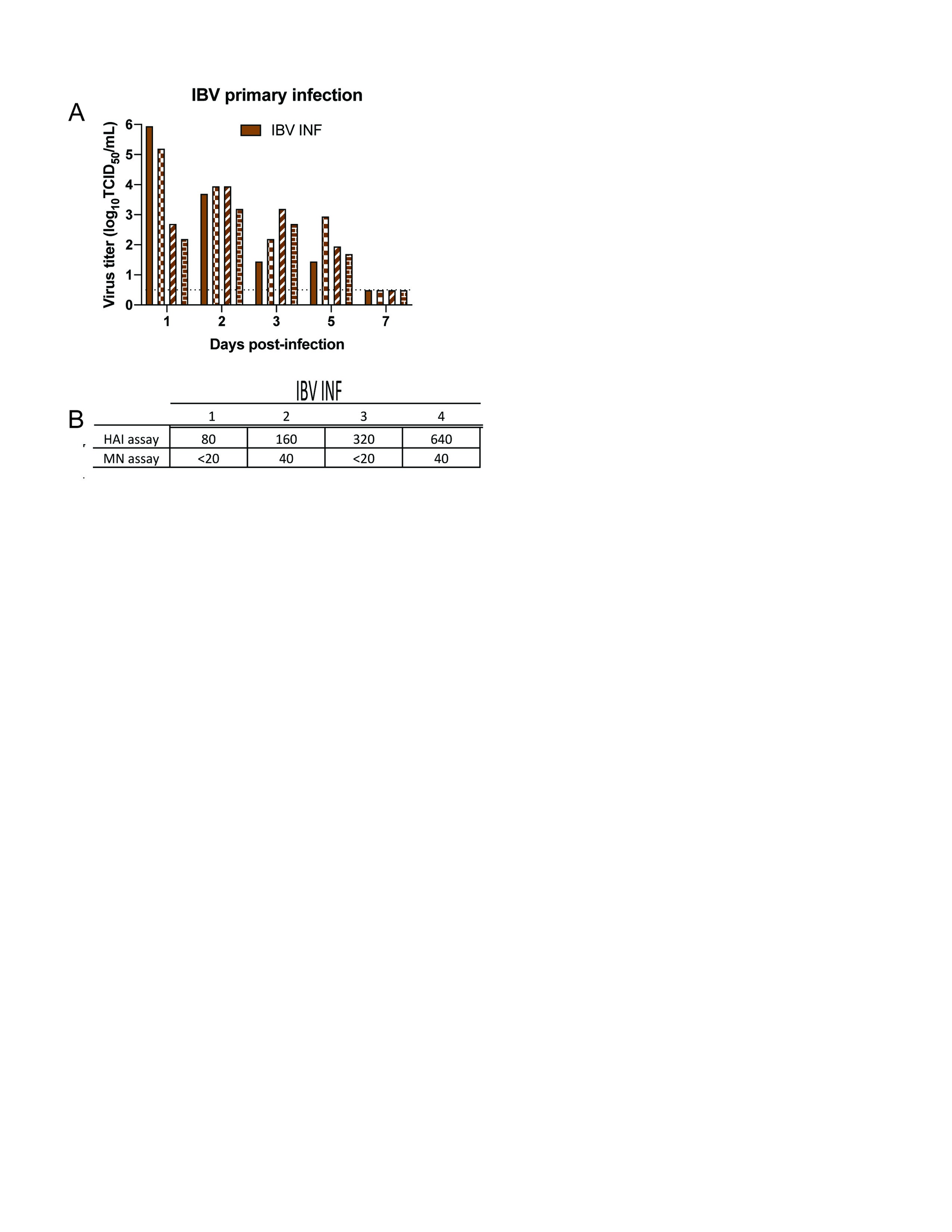

Supplement: S4 Fig — (A) Four ferrets were infected with B/Brisbane/60/2008 and nasal washes were collected from each ferret on the indicated days post-infection. Bars indicate individual ferrets. The limit of detection is represented by the dashed line. (B) All ferrets were confirmed to be serologically negative for circulating influenza A and B viruses at the beginning of the study. The presence of influenza B antibodies on day 14 post-infection was detected in all infected animals by HAI, but neutralization titers were only observed in 2/4 infected ferrets. TCID50, 50% tissue culture infectious dose. HAI, hemagglutination inhibition. MN, microneutralization. (TIF) [file ppat.1009273.s004.tif]

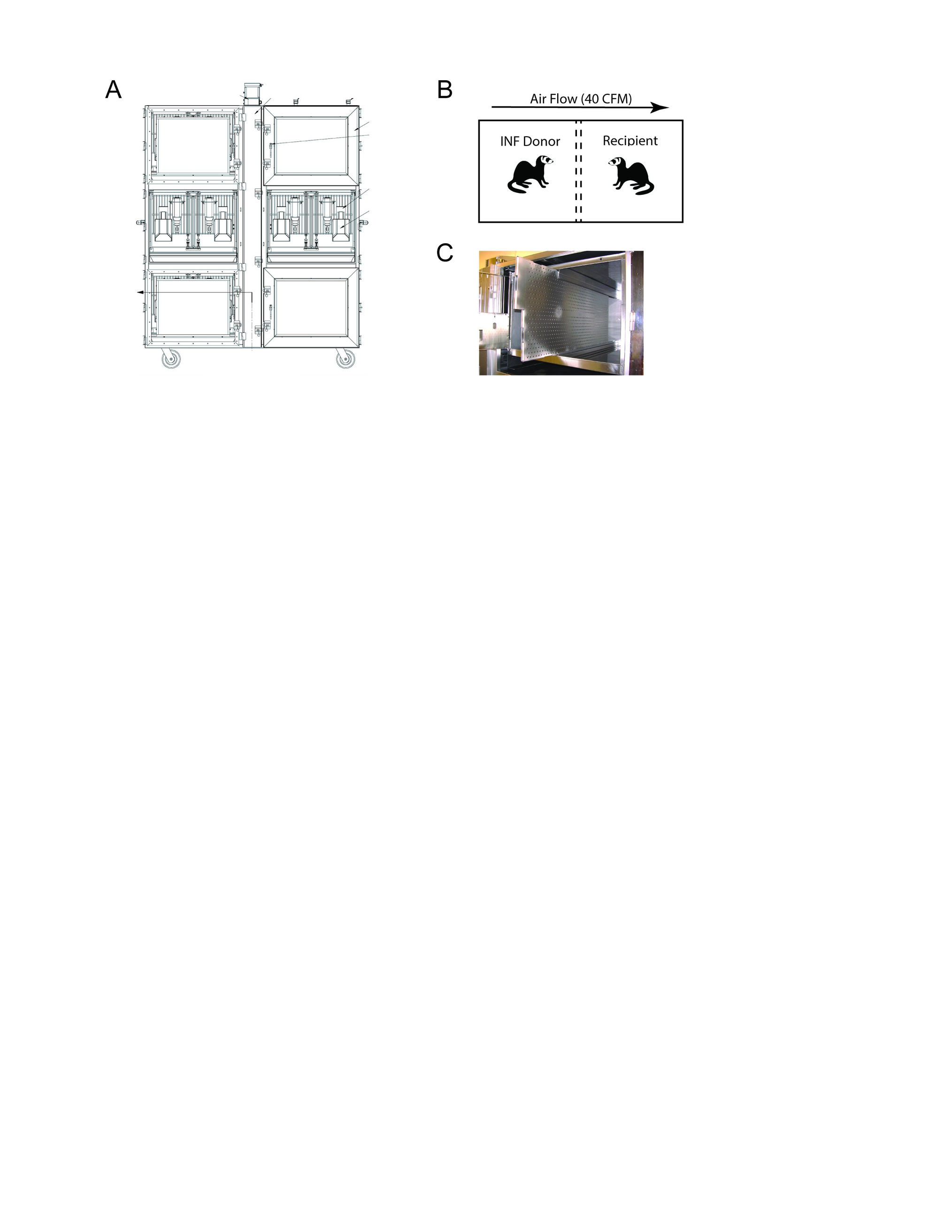

Supplement: S5 Fig — A. Diagram of ferret transmission unit. B. Schematic of ferret experimental setup with constant air flow passing from infected (INF) donor to recipient ferret. The rack has a flow rate of 40 cubic feet per minute (CFM), for 35 air changes per hour within the total rack. INF donor and recipient are separated by a stainless steel divider, which is made up of two perforated plates with 5mm diameter holes, the plates are welded together 2 cm apart such that the holes are staggered. (TIF) [file ppat.1009273.s005.tif]

**Table S1.** Clinical signs and symptoms.


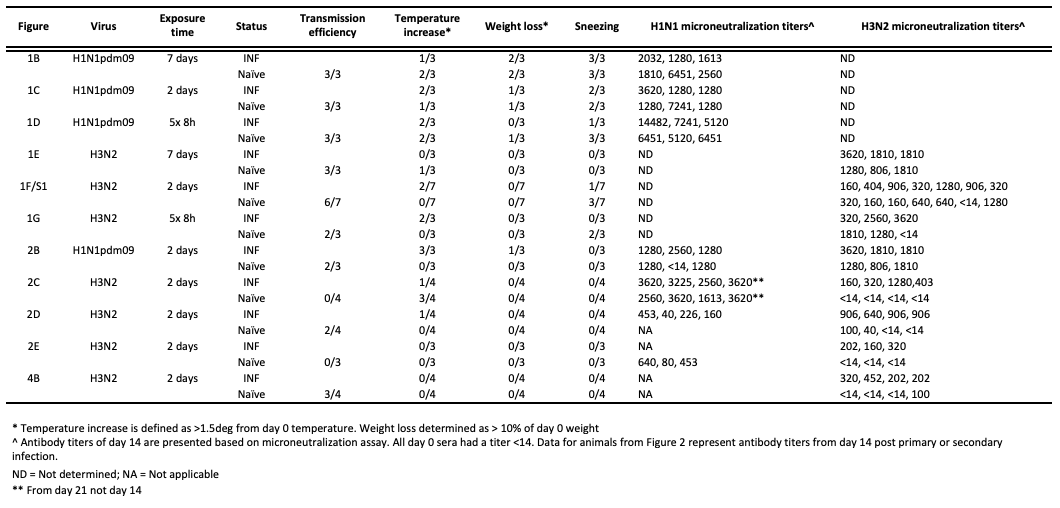

Supplement: S1 Table — (DOC) [file ppat.1009273.s006.doc]
