# Supplementary material for: Pre-existing heterosubtypic immunity provides a barrier to airborne transmission of influenza viruses
Source: PLoS Pathog. 2021 Feb 18;17(2):e1009273. doi: 10.1371/journal.ppat.1009273 (PMC7891786; doi:10.1371/journal.ppat.1009273)
Supplement: Attachment — Submitted filename: rebuttal_201116_ssl_201127.docx [file ppat.1009273.s007.docx]

Reviewer's Responses to Questions

***We thank the reviewers for their comments on our manuscript. A point-by-point response to the criticisms are appended below.*** *Author responses are in blue. Modified text is highlighted on marked up text.*

Reviewer #2: My major concern is with regards to the lack of a potential mechanism underlying the limited transmission in immune animals, and why that differs between H1 and H3 infection/transmission events. The lack of neutralising antibodies between H1 and H3 viruses is not particularly surprising, neither is the lack of any innate effects, given that long period between primary and secondary infections. Any additional data (eg HA stalk titers, NA antibodies) that may be available could provide additional insights into this. At the very least, I think the discussion could benefit from some additional points on what maybe limiting transmission. It is pertinent to also mention prior evidence for:
- HA stalk antibodies can reduce susceptibility to transmission (eg ref 36 of manuscript)
- NA antibodies can limit transmission of IBV in a guinea pig model (MacMahon et al 2019, 10.1128/mBio.00560-19)
- CD8+ T cells are known to confer broad cross-protection between heterosubtypic infections and may explain some of the observations of reduced disease severity as mentioned in discussion lines 278-280.

*We thank the reviewer for this comment and agree that antibodies against HA stalk or NA can impact transmission efficiency. We have addressed this in the manuscript in two ways. First, previous work we published recently examined the induction of group 1 and 2 stalk antibodies in ferrets after primary H1N1 or H3N2 infection and again after secondary infection with the heterosubtypic virus. The work in Arevalo et al PNAS complements our current study and demonstrated that primary H1N1pdm09 infection does not produce detectable group 2 stalk antibodies after primary infection (Fig 1D in Arevalo et al PNAS PMID: 32631992). Therefore, cross-reactive stalk antibodies induced after H1N1pdm09 infection are not likely to be the mechanism of protection although there may be contribution of low levels of cross-reactive stalk antibodies. We have added text to address this on lines 296-299 (highlighted on the marked up version).*

*To examine the presense of cross-reactive NA antibodies, we measured N1 and N2 specific antibodies using an ELISA on purified N1 and N2 proteins. This new data is presented in Supplemental Figure 3, and demonstrates that the infection with H1N1pdm09 did not induce detectable cross-reactive NA antibodies. See text in results section on lines 209-222 (highlighted on marked up version). We have extended the discussion to include NA antibodies, as well as other potential mechanisms of protection please see lines 300-323.*

Reviewer #3: The current studies are technically sound; however, the high infection dose likely overwhelmed the potency of pre-existing immunity. The authors should discuss whether prior reports provide evidence of cross-protective cellular immunity. *Pre-existing immunity induced by IBV infection was a good control experiment, but it does not provide suggestive data as to why H3N2 prior immunity failed to protect against infection by airborne transmitted H1N1 influenza virus*. It is also unclear how efficiently IBV replicated in ferrets, and thus established pre-existing immunity.

*We thank the review for these comments. To address many of the concerns raised, we have added details into the discussion regarding cross-protective immunity, please see lines 313-323. We have added the virus titer data for the ferrets infected with IBV, which replicated to high titers (see new Supplemental Fig 4A). IBV immunity was determined on day 14 post-infection by HAI and MN, the titers are now presented in Supplemental Fig 4B.*

*We agree that the the inoculation dose of 10^6^ TCID_50_ in 500uL of influenza virus is higher than the dose needed to initiate an infection in ferrets or humans. However, recent dose finding studies in human volunteers have demonstrated that 10^7^ TCID_50_ of 2009 H1N1pdm virus was needed to infect 69% of volunteers and produced mild symptoms (Memoli et al Clinical Infect Disease 2015 PMID:25416753). In addition, a similar dose escalation study for H3N2 viruses suggested the need for a dose of 10^6^ or 10^7^ TCID_50_ to infect >40% of humans (Han et al Clinical Infection Disease 2019 PMID:30770534). These high doses for human challenge studies are likely needed to overcome a pre-existing immunity barrier to produce a mild symptomatic infections. This revised human infectious dose is on par with our challenge dose for animals with pre-existing immunity to ensure a robust infection.*

**Part III – Minor Issues: Editorial and Data Presentation Modifications**
Please use this section for editorial suggestions as well as relatively minor modifications of existing data that would enhance clarity.

Reviewer #1: -line 78, would this repeated exposure not be inclusive of both infection and vaccination? Overall throughout the study, it would be of benefit for the authors to clarify and expand upon if they believe vaccination would elicit comparable results, or if their findings are specific to immunity acquired via live virus infection.

*We thank the reviewer for this suggestion and agree that the impact of vaccination on transmission is a very interesting avenue of future research but is outside the scope of this article. We have added a paragraph to the discussion regarding vaccination on lines 324-330.*

-line 110, “a representative seasonal H3N2 influenza virus, A/Perth/16/2009” – how antigenically related is this strain to the currently circulating virus clades?

*We modified this sentence to be ‘a representative human H3N2 influenza virus from the same time period’ on line 112 to acknowledge that current circulating strains are antigenically distinct from the 2009 H3N2 virus.*

-Figure 1A/S3, it’s currently unclear if the contact ferrets that had intermittent exposure to donor ferrets (8hr/day for 5 days) were removed from their cage outright outside of these exposure windows to a clean cage that was free of virus, or if a solid barrier was placed at the perforated stainless steel interface separating inoculated and donor ferrets to abrogate exposure to new aerosols shed from donor ferrets (while maintaining exposure to any virus still present in the cage), please specify.

*We have included additional details to the materials and methods section on lines 771-774.*

-Lines 175-78/296-8: Do the authors know the relative 50% ferret infectious dose for the H1N1 and H3N2 viruses employed here? Is it possible that higher levels of exhaled infectious virus from the H1N1 donor ferrets and/or increased transmissibility at lower exposure levels is contributing to the subtype-specific profiles of transmission presented in experiments from Figure 2 (i.e. that the H1N1 but not H3N2 virus is overcoming preexisting heterosubtypic immunity in part because of higher viral exposures in the contact animals)?

*Response: A dose of 10^2^ TCID_50_ in 500uL of H1N1pdm09 results in virus shedding and seroconversion of 100% of the ferrets infected (Lakdawala et al., 2011 PMID: 22241979). To determine the infectivity of H3N2 (A/Perth/16/2009), we infected naïve ferrets with 10^2^ TCID_50_ in 500uL, which resulted in 0/3 ferrets shedding or seroconverting. Therefore, the FID_50_ for H3N2 is likely different than H1N1pdm09, but to conclusively make this case, additional ferret studies are required and are tentatively planned for next year. However, we believe this is beyond the scope of this current manuscript and plan to include the data in a future manuscript. We have provided discussion about the potential impact of pre-existing immunity on ferret infectious dose on lines 273-283.*

-Lines 190-92: Nasal washes capture relative levels of virus replication in the ferret respiratory tract but are not necessarily a strong surrogate for virus shed into the environment from inoculated animals; consider changing “…not due to significantly decreased viral shedding” to “…viral replication” (or otherwise clarify the statement to account for this).

*The statement has been modified.*

Reviewer #2: - The manuscript might benefit from adding ‘heterosubtypic’ to the title. eg “Pre-existing heterosubtypic immunity provides a barrier….”

*We have added ‘heterosubtypic’ to the title.*

- The abstract states (lines 37-38) “viruses are highly transmissible to naïve animals within 48 hours”. This may be misinterpreted to mean that the viruses were sown to be transmitted within the first 48 hours of infection, while the 48 hour window was from 24-72 hours according to fig 1A. Can this statement be rephrased to something like “a 48 hour exposure is sufficient for efficient transmission”

*These sentences have been modified.*

- Sup table 1 states that animals in fig 2B were exposed for 7 days, while the schematic in fig 2A shows a 2 day exposure. Can the authors please clarify and amend accordingly.

*We thank the reviewer for catching this error and have corrected supplemental table 1.*

Reviewer #3: How pre-existing immunity against infection by transmitted influenza virus is a potential correlate of protection is unclear and should be explained with more detail. The authors indicated prior studies that examined this topic, but it is unclear how the current experimental design improves upon these previous studies. The authors indicated differences between air flow rates, but it is unclear if liters per minute are similar to or substantially divergent from cubic feet per minute.

*Discussion into the potential correlates of protection has been added please see lines 289-323. We agree that liters per minute and cubic feet per minute are hard to compare and air changes per hour (ACH) is a better source of comparison between caging systems, however the dimensions for the Koster et al caging setup are not available and so we are not able to calculate the precise ACH between both systems. We have modified the text to remove the ambiguity between air flow rates. Rather we acknowledge that all transmission systems are variable which can influence the relative transmission efficiency observed at short exposure times (lines 269-271).*
